# Prevalence of Dental Fluorosis and Its Association With Body Mass Index Among School-Age Children in Hail, Saudi Arabia: A Cross-Sectional Study

**DOI:** 10.7759/cureus.110033

**Published:** 2026-06-01

**Authors:** Omar A Almohsen, Ali Alsweed

**Affiliations:** 1 Pediatric Dentistry, Private Practice, Bukayriyah, SAU; 2 Pediatric Dentistry, Ministry of Health, Qassim Region, Buraydah, SAU

**Keywords:** body mass index, children, fluorosis, permanent dentition, primary dentition

## Abstract

Dental fluorosis, characterized by enamel hypomineralization due to excessive fluoride ingestion during the tooth development stage, remains a critical public health challenge in regions where groundwater contains high natural fluoride concentrations. A cross-sectional analysis was carried out including 524 schoolchildren aged 9-12 years in Hail, Saudi Arabia. The findings demonstrated a substantial prevalence of dental fluorosis, with a highly significant difference observed between primary and permanent dentitions and BMI categories (p < 0.0001). However, statistical analysis revealed no significant association between fluorosis occurrence and gender, whether considered overall or within specific dentition types. These results suggest that while dentition type and BMI are strong predictors of fluorosis prevalence, gender may not be the primary determinant in this population. The study emphasizes the necessity for integrated public health strategies that address environmental fluoride exposure in high-fluoride areas.

## Introduction

Dental fluorosis is a developmental anomaly of the enamel arising from chronic exposure to elevated fluoride levels during the formative stages of tooth development. Clinically, it presents as a spectrum of manifestations, ranging from inconspicuous white striations to pronounced brownish discoloration and structural pitting. The severity of these enamel defects is fundamentally determined by the dosage, duration, and specific timing of fluoride intake [1]. The Hail region of Saudi Arabia is particularly recognized for its high natural fluoride content in groundwater, which has historically contributed to an endemic prevalence of dental fluorosis within the local population [2].

On a global scale, dental fluorosis and pediatric obesity, typically evaluated via body mass index (BMI), constitute major public health concerns. While the nexus between dental caries and BMI has been extensively scrutinized, often yielding divergent conclusions [3,4], the relationship between dental fluorosis and BMI remains less clearly defined. Emerging evidence suggests a complex interplay; some studies indicate that children with lower BMI may exhibit increased vulnerability to fluorosis [5], whereas other studies have identified positive associations between fluoride exposure and adiposity in specific cohorts [6,7].

Given the endemic nature of fluorosis in Hail and the rising global trends in childhood weight abnormalities, investigating the potential association between these two conditions in this specific demographic is vital for refining public health interventions. Thus, this study aims to elucidate the prevalence of dental fluorosis and its relationship with BMI and gender among school-age children.

## Materials and methods

To investigate the prevalence of fluorosis and its correlation with BMI, a cross-sectional survey was performed among 524 students aged 9-12 years in Hail, Saudi Arabia. The study was conducted over two months from December 2019 to January 2020. The study was approved by the Dental Ethics Committee, Qassim University (approval #DRC/003FA/19).

Stusy population

The sampling strategy involved a cluster-based approach, utilizing the city's three administrative districts as primary units. Within each district, three schools were selected via simple random sampling. Eligible participants were enrolled following a comprehensive briefing on the study's objectives and the acquisition of written informed consent from both the children and their legal guardians.

For a target population of 20,000 children (ages 9-12) in the Hail region, Saudi Arabia, a sample size of 377 was calculated using a 95% confidence level and a 5% allowable error. By examining all consenting, healthy children present at the participating schools, the final sample was expanded to 524. To ensure data integrity, the study excluded any children with medical conditions that could impact dental development or enamel integrity.

Data collection

Following the recording of socio-demographic profiles and anthropometric measurements, clinical dental assessments were carried out under standardized natural daylight conditions. To ensure procedural precision, participants were evaluated individually in an upright chair. A single-examiner protocol was strictly followed to prevent inter-examiner variability, with a trained recorder assisting in the process. Intra-examiner consistency was rigorously tested via a pre-study calibration involving 25 children examined twice over a two-day period, resulting in a high Kappa score of >0.87. 

Dental fluorosis was recorded as per the criteria of Dean’s Index [8]. Participants' weight was measured to the nearest 0.1 kg (GS58-1694 electronic scale; Beurer GmbH, Ulm, Germany) without shoes and in light attire. Height was assessed to the nearest full centimeter using a Helsevesen free-standing stadiometer in the standing position. BMI was computed using the standard formula: weight (kg)/ height² (m²).

Statistical analysis

The analysis utilized a dataset containing fluorosis status (normal vs. any fluorosis) categorized by BMI (Underweight, Normal weight, Overweight), gender (Male, Female), and dentition type (Primary, Permanent). Chi-square tests of independence were performed to assess the statistical significance of associations between categorical variables. For each comparison, a contingency table was constructed, tallying the counts of individuals with and without fluorosis across the respective categories. A p-value less than 0.05 was considered statistically significant. IBM SPSS Statistics for Windows, version 26.0 (IBM Corp., Armonk, New York, United States) was used for statistical analysis.

Odds Ratios (OR) and their 95% Confidence Intervals (CI) were calculated to quantify the strength and direction of associations. An OR greater than 1 indicates an increased odds of fluorosis in the exposed group compared to the reference group, while an OR less than 1 indicates decreased odds. The 95% CI provides a range within which the true OR is likely to fall.

## Results

A total of 524 children were examined, of whom 329 students (62.8%) were male, and 195 (37.2%) were female. One hundred forty-six students were underweight, 229 were of normal weight, and 149 were overweight. The overall prevalence of fluorosis in primary dentition was 10.11% (n=53), and in permanent dentition, it was 23.09% (n=121).

**Figure 1:**
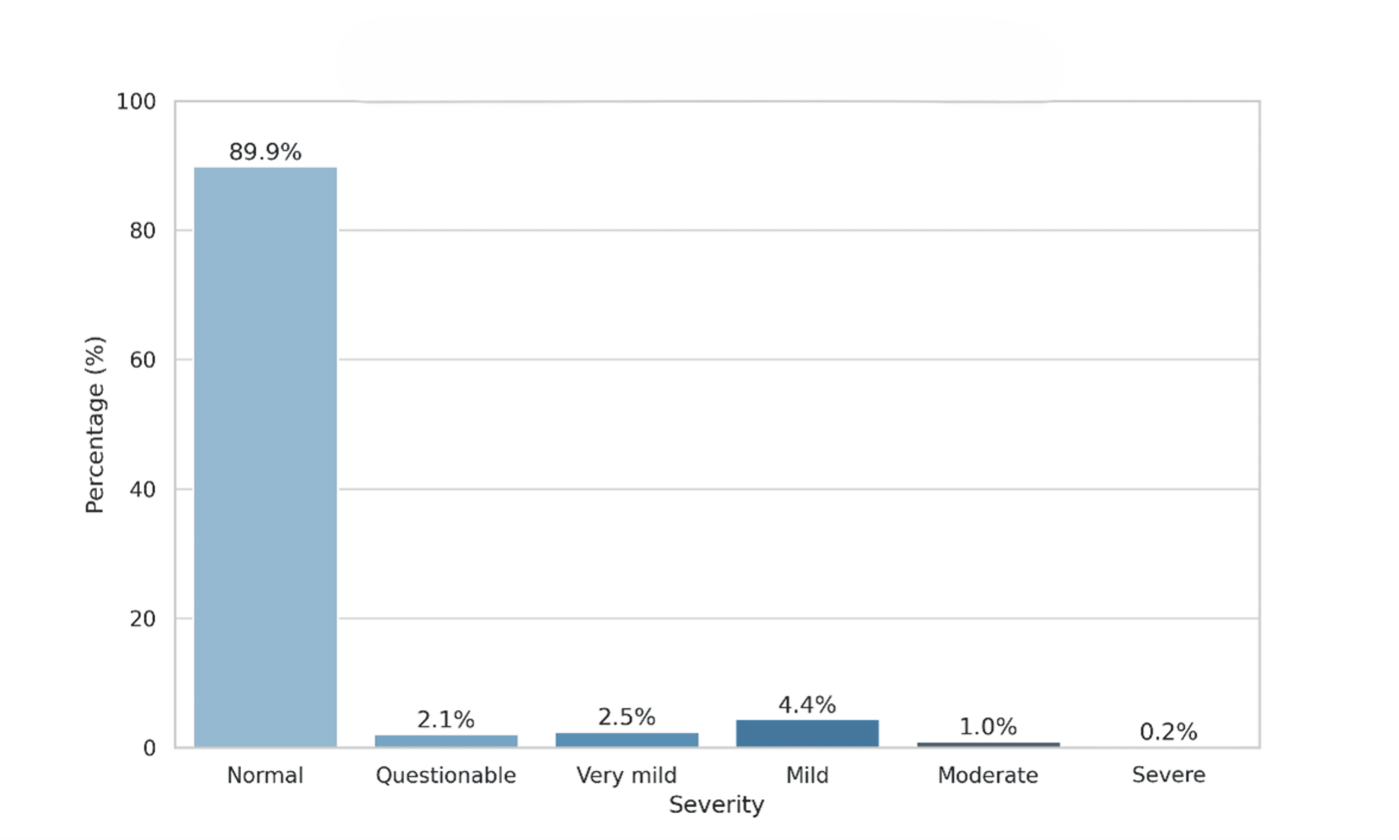
Severity of fluorosis within primary dentition.

**Figure 2:**
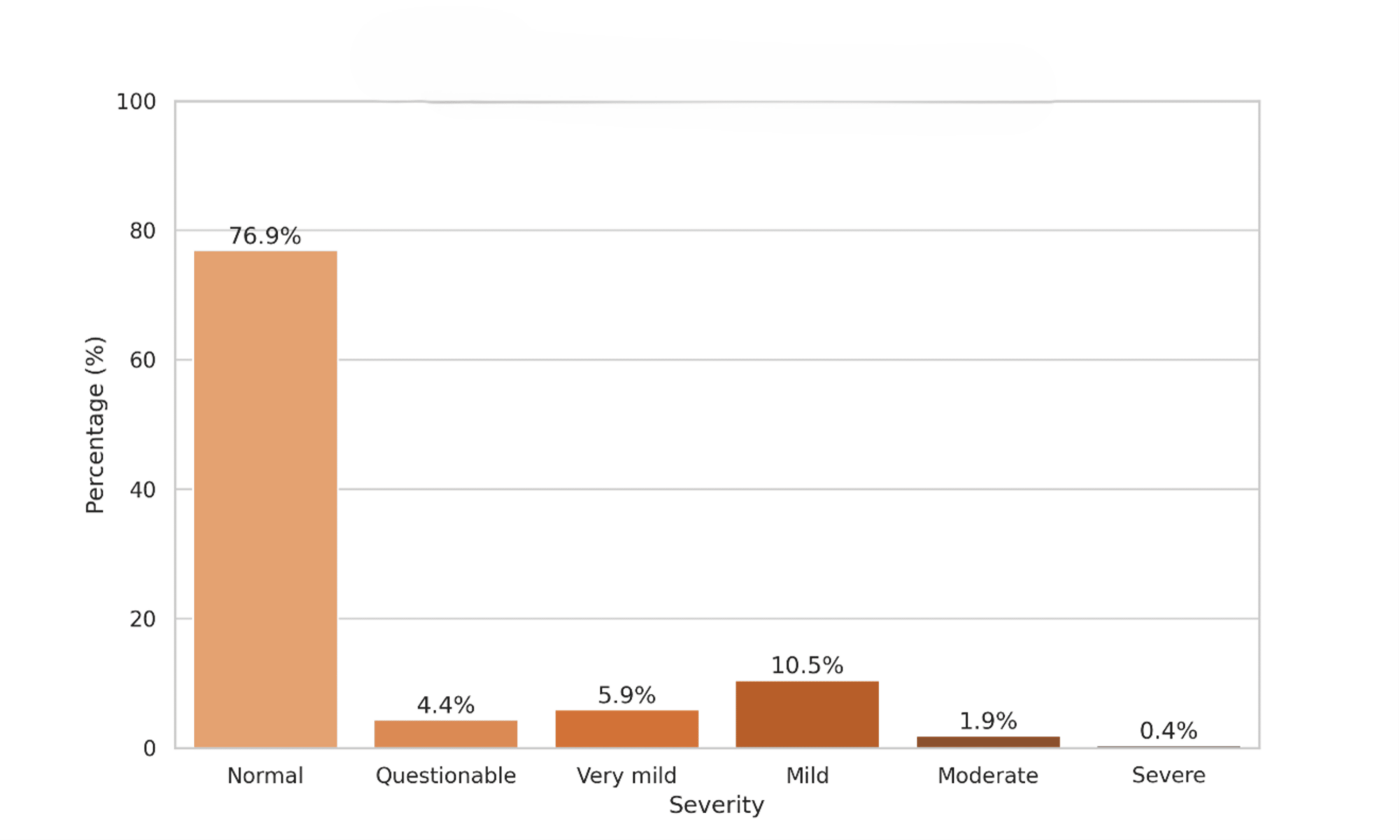
Severity of fluorosis within permanent dentition.

The severity of fluorosis recorded in primary dentition was 2.1%, 2.5%, 4.4%, 1%, and 0.2% as classified in Dean's index, which were questionable, very mild, mild, moderate, and severe, respectively (Figure 1). In permanent dentition, the severity was 4.4%, 5.9%, 10.5%, 1.9%, and 0.4%, denoting very mild, mild, moderate, and severe, respectively (Figure 2).

The prevalence of fluorosis was not affected by gender in primary dentitions with no statistically significant difference (p=0.6813) (Table 1). Similar result observed in permanent dentition (p=0.5876) (Table 2).

**Table 1:** Association of gender with fluorosis in primary dentition Pearson chi-square test, χ²(1) = 0.0069, p = 0.9339. Male was used as the reference category for ratio estimates.

Gender	Fluorosis absent, n	Fluorosis present, n	Total	Fluorosis prevalence, % (95% CI)	Prevalence ratio (95% CI)	Odds ratio (95% CI)	p-value
Female	175	20	195	10.3% (6.7–15.3)	1.02 (0.60–1.73)	1.03 (0.57–1.84)	0.9339
Male	296	33	329	10.0% (7.2–13.8)	Reference	Reference	0.9339

**Table 2:** Association of gender with fluorosis in permanent dentition Pearson chi-square test, χ²(1) = 0.4219, p = 0.5160. Male was used as the reference category for ratio estimates.

Gender	Fluorosis absent, n	Fluorosis present, n	Total, n	Fluorosis prevalence, % (95% CI)	Prevalence ratio (95% CI)	Odds ratio (95% CI)	p-value
Female	153	42	195	21.5% (16.3–27.8)	0.90 (0.65–1.25)	0.87 (0.57–1.33)	0.5160
Male	250	79	329	24.0% (19.7–28.9)	Reference	Reference	0.5160

There was a statistically significant association between BMI category and fluorosis status in primary dentition (p < 0.001). Fluorosis was much more common among underweight children compared with normal-weight and overweight children. The overweight group did not show a higher risk; it was similar to or lower than that of the normal-weight group (Table 3). Similar results were also observed in permanent dentition with a statistically significant difference (p < 0.001) (Table 4).

**Table 3:** Association of BMI category with fluorosis in primary dentition Pearson chi-square test, χ²(2) = 43.9203, p < 0.001. Cramer's V = 0.2895. Normal weight was used as the reference category for ratio estimates.

BMI	Fluorosis absent, n	Fluorosis present, n	Total	Fluorosis prevalence, % (95% CI)	Prevalence ratio (95% CI)	Odds ratio (95% CI)	p-value
Normal weight	215	14	229	6.1% (3.7–10.0)	Reference	Reference	<0.001
Overweight	145	4	149	2.7% (1.0–6.7)	0.44 (0.15–1.31)	0.42 (0.14–1.31)	<0.001
Underweight	111	35	146	24.0% (17.8–31.5)	3.92 (2.19–7.03)	4.84 (2.50–9.38)	<0.001

**Table 4:** Association of BMI category with fluorosis in permanent dentition Pearson chi-square test, χ²(2) = 74.5109, p < 0.001. Cramer's V = 0.3771. Normal weight was used as the reference category for ratio estimates.

BMI	Fluorosis absent, n	Fluorosis present, n	Total	Fluorosis prevalence, % (95% CI)	Prevalence ratio (95% CI)	Odds ratio (95% CI)	p-value
Normal weight	197	32	229	14.0% (10.1–19.1)	Reference	Reference	<0.001
Overweight	131	18	149	12.1% (7.8–18.3)	0.86 (0.50–1.48)	0.85 (0.46–1.57)	<0.001
Underweight	75	71	146	48.6% (40.7–56.7)	3.48 (2.42–5.00)	5.83 (3.55–9.56)	<0.001

In comparison between primary and permanent dentition, there was a statistically significant association between dentition type and fluorosis (p < 0.0001) (Table 5). The odds of having fluorosis were 2.67 times higher in permanent dentition compared to primary dentition, with a 95% CI of 1.88 to 3.78, indicating a robust and significant difference.

**Table 5:** Association of dentition type with fluorosis Pearson chi-square test using marginal dentition-type counts, χ²(1) = 31.8653, p < 0.001. Primary dentition was used as the reference category for ratio estimates.

Dentition type	Fluorosis absent, n	Fluorosis present, n	Total observations, n	Fluorosis prevalence, % (95% CI)	Prevalence ratio (95% CI)	Odds ratio (95% CI)	p-value
Primary dentition	471	53	524	10.1% (7.8–13.0)	Reference	Reference	<0.001
Permanent dentition	403	121	524	23.1% (19.7–26.9)	2.28 (1.69–3.08)	2.67 (1.88–3.78)	<0.001

## Discussion

This study aimed to investigate the prevalence of dental fluorosis and its association with gender and BMI categories among school-age children in Hail, Saudi Arabia, with a particular focus on distinguishing findings between primary and permanent dentition.

Consistent with established literature and the known high fluoride content in Hail's groundwater [1,2,8-11], our findings confirm a substantial endemic prevalence of dental fluorosis in the study population. A highly significant difference in fluorosis prevalence was observed between primary and permanent dentition, with permanent teeth exhibiting approximately 2.67 times higher odds of fluorosis compared to primary teeth. This disparity is biologically plausible and aligns with the understanding that permanent teeth have a longer developmental period, extending into childhood, which increases their cumulative exposure to fluoride during critical stages of enamel formation and maturation [8].

The WHO Guidelines for Drinking-water Quality report underscores fluoride's complex role. It acknowledges that appropriate fluoride levels are vital for safeguarding against tooth decay. However, it is also cautions that elevated fluoride concentrations can lead to adverse health outcomes, notably dental and skeletal fluorosis [12].

Primary teeth, forming predominantly in utero and early infancy, are exposed to fluoride for a shorter duration and often at lower systemic levels, thus generally exhibiting lower fluorosis rates. A similar result was found as fluorosis in primary dentition is less common [13]. While dental fluorosis in the primary dentition is infrequent, its occurrence is predominantly localized to the posterior teeth, specifically the primary second molars. Given that these teeth undergo mineralization at a later developmental stage, this pattern suggests that primary tooth fluorosis is largely a postnatal event, significantly influenced by elevated fluoride concentrations in the water supply [13].

Contrary to some initial hypotheses and qualitative observations, our rigorous statistical analysis found no significant association between gender and the presence of fluorosis, either when considering the overall population or when analyzed independently within primary and permanent dentition. Gender does not appear to be a significant determinant of fluorosis risk in this study.

However, the analysis revealed a statistically significant association between BMI categories and fluorosis prevalence within primary and permanent dentition.

Limitations

This study, being cross-sectional, provides a snapshot of the association between fluorosis and the studied factors at a single point in time. It cannot establish causality. The study also relied on Dean's Index for fluorosis assessment, which is a clinical index and does not directly measure fluoride intake levels. Future research could benefit from longitudinal designs, more detailed assessments of fluoride exposure, and comprehensive nutritional evaluations

## Conclusions

This study shows difference in fluorosis prevalence between primary and permanent dentition among school-age children in Hail, Saudi Arabia, highlighting the greater susceptibility of permanent teeth to fluorosis. These findings suggest that while dentition type and BMI is a strong predictor, gender may not be primary determinants of fluorosis risk in this population. The study underscores the importance of continued public health efforts to mitigate fluoride exposure in endemic areas, with a focus on the developmental stages of permanent dentition. Further research is warranted to explore other potential modifying factors and to understand the long-term implications of fluorosis in this region.
